# Pregnancy at early age is associated with a reduction of progesterone-responsive cells and epithelial Wnt signaling in human breast tissue

**DOI:** 10.18632/oncotarget.16023

**Published:** 2017-03-08

**Authors:** Simone Muenst, Robert Mechera, Silvio Däster, Salvatore Piscuoglio, Charlotte K.Y. Ng, Fabienne Meier-Abt, Walter P. Weber, Savas D. Soysal

**Affiliations:** ^1^ Institute of Pathology, University Hospital Basel, Basel, Switzerland; ^2^ Department of Surgery, University Hospital Basel, Basel, Switzerland; ^3^ Department of Biomedicine, University Hospital Basel, Basel, Switzerland; ^4^ Institute of Hematology, University Hospital Zurich, Zurich, Switzerland

**Keywords:** early pregnancy, breast cancer, Wnt signaling pathway, breast cancer prevention, Pathology Section

## Abstract

**Background:**

Pregnancy at early age is the most significant modifiable factor which consistently decreases lifetime breast cancer risk. However, the underlying mechanisms haven't been conclusively identified. Studies in mice suggest a reduction in progesterone-receptor (PR) sensitive epithelial cells as well as a downregulation of the Wnt signaling pathway as being one of the main mechanisms for the protective effect of early pregnancy. The aim of our study was to validate these findings in humans.

**Methods:**

We collected benign breast tissue of 125 women who had been stratified according to age at first pregnancy and the occurrence of subsequent breast cancer, and performed immunohistochemistry for PR, Wnt4 and the Wnt-target Versican.

**Results:**

The number of PR positive epithelial cells was significantly lower in the group of women with early pregnancy and no subsequent breast cancer compared to the group of nulliparous women with subsequent invasive breast cancer (*p* = 0.0135). In women with early pregnancy, expression of Versican and Wnt4 was significantly lower compared to nulliparous women (*p* = 0.0036 and *p* = 0.0241 respectively), and Versican expression was also significant lower compared to women with late pregnancy (*p* < 0.0001).

**Discussion:**

Our results confirm prior observations in mice and suggest a role of downregulation of epithelial Wnt signaling in the protective effect of early pregnancy in humans. This results in a decreased proliferation of stem/progenitor cells; therefore, the Wnt signaling pathway may represent a potential target for breast cancer prevention in humans.

## INTRODUCTION

With an estimated 1.7 million cases per year worldwide, breast cancer is the most frequent cancer in women. Despite substantial progress in early detection and treatment, 521.900 women die of their disease each year [1]. Several risk factors for breast cancer have been described. One of the strongest modifiable factors which consistently decreases lifetime breast cancer risk, is pregnancy [2–4].

The protective effect of a full-term pregnancy is most pronounced for a pregnancy occurring before the age of 20, resulting in a lifetime breast cancer risk reduction of >50% in comparison to nulliparous women [2]. While the protective effect becomes negligible for women with first full-term pregnancies between the age of 30 and 34, an increased risk can be observed in women with a first pregnancy over the age of 35 [5]. Interestingly, a transient increase in breast cancer risk can be observed in women over the age of 25 years immediately after delivery, delaying the protective effect by up to 10 years [3]. In addition to early age at first pregnancy, multiple pregnancies as well as prolonged breast feeding also decrease the life-time risk for breast cancer to a smaller degree [6]. It seems that parity specifically protects against hormone receptor positive breast cancers, whereas the risk for hormone receptor negative cancers is not influenced by age at first pregnancy or parity [7]. The effect of pregnancy-induced protection against breast cancer has also been observed in rodent models [8, 9].

The underlying mechanisms of this protective effect have not been conclusively identified and may not be explainable by a solitary mechanism, but by a combination of mechanisms [6] such as *alterations in circulating hormones* [6, 8], a parity induced *decrease of hormone responsiveness* of the mammary gland [6, 8], long-lasting *alterations in the composition of the mammary extracellular matrix (ECM)* [6, 8, 10], as well as a *terminal epithelial cell differentiation* occurring during pregnancy [6, 11–13].

Parity induced *alteration of cell fate of specific mammary epithelial cells* including mammary stem/progenitor cells represents another promising explanation. Persistent changes in transduction pathways, growth and transcription factors and/or cell cycle regulatory molecules during pregnancy may allow breast epithelial cells to become less proliferative and more resistant to carcinogenesis [6, 14]. Of particular significance are mammary stem/progenitor cells which represent an ideal target for malignant transformation due to their self-renewing capacity and longevity [15, 16].

Meier-Abt et al. [17] analyzed the cell fate hypothesis in various mammary epithelial cell subpopulations in mice. In this study, mammary glands of parous and virgin mice were harvested, and gene-expression profiles and differentiation/proliferation potentials of specific mammary subpopulations were analyzed. In addition, the mammary glands of parous and age matched virgin control mice were immunohistochemically analyzed for the expression of estrogen receptor α-, progesterone receptor (PR), Versican, and β-catenin. The Versican gene encodes for an extracellular proteoglycan and represents one of the classic target genes of the Wnt pathway [17].

The results of their study suggested that in mice, early pregnancy leads to a reduced response of the mammary gland to progesterone, caused primarily by a decrease in the proportion of PR positive luminal cells. Consecutively, a downregulation of Wnt4 expression in the luminal epithelial cells, and a reduction of the Wnt signaling pathway in basal stem/progenitor cells was identified [17]. Wnt ligands, such as Wnt4 in breast, activate a signaling cascade of which the Wnt/β-catenin-dependent pathway is best characterized [18, 19]. Activation of the Wnt signaling pathway causes accumulation of β-Catenin in the nucleus, resulting in an interaction with transcription factors and expression of the respective target genes [18–20], which has been shown to promote tumorigenesis in mice [21] and promotes invasiveness of human breast cancer cells by interacting with the Hippo pathway [22]. On the other hand, downregulation of Wnt- signaling causes a persistent decrease of proliferation potential of basal mammary stem/progenitor cells and an increased differentiation phenotype of mammary epithelial cells [17]. These findings suggest that the Wnt signaling pathway plays a crucial role in breast cancer carcinogenesis, which might be mediated by increased proliferation of mammary stem/progenitor cells [6].

In a second study, the authors demonstrated that the reduction of PR expressing cells and downregulation of Wnt signaling pathway in parous mice are of life-long duration [23], and that pregnancies in older animals have a limited effect on these parameters [23].

In humans, only a few studies have analyzed the effect of pregnancy on breast cancer cells. A decrease of PR positive luminal cells as well as Wnt signaling can also be observed in parous compared to nulliparous women [24, 25]. However, to our knowledge, a systematic analysis of the Wnt signaling pathway as well as PR expression in women with pregnancy at an early age compared to women with pregnancy at an older age and nulliparous women has not been conducted so far. The aim of the present study was to analyze whether the same mechanisms as described in mice might also play a role in the protective effect of early pregnancies against breast cancer in humans, in order to identify potential targets for breast cancer prevention strategies.

## RESULTS

Of the 125 patients identified, 35 (28%) were nulliparous, 52 (42%) had full-term pregnancy before the age of 30 and 38 (30%) after the age of 30 (Table 1). Within the nulliparous group, 23 (66%) did not have subsequent breast cancer (Group 1) and 12 (33%) developed subsequent breast cancer (Group 2). Within the group of women that had full-term pregnancy before the age of 30, 16 (31%) developed subsequent breast cancer (Group 3) whereas 36 (69%) did not have subsequent breast cancer (Group 4). Within the group of women that had full-term pregnancy after the age of 30, 16 (42%) developed subsequent breast cancer (Group 5) and 22 (58%) did not have subsequent breast cancer (Group 6).

**Table 1:** Number of subjects and median age in each group based on age at first pregnancy and the occurrence of subsequent breast cancer

Groups	Number of patients	Age at first pregnancy (median)	Age at cancer diagnosis (median)
1 (nulliparous, no subsequent cancer)	25	-	-
2 (nulliparous, subsequent cancer)	10	-	35-79 (51)
3 (early pregnancy, subsequent cancer)	16	18-29 (23)	34-71 (52)
4 (early pregnancy, no subsequent cancer)	36	18-27 (21)	-
5 (late pregnancy, subsequent cancer)	16	33-38 (36)	39-69 (56)
6 (late pregnancy, no subsequent cancer)	22	32-39 (37)	-

**Figure 1:**
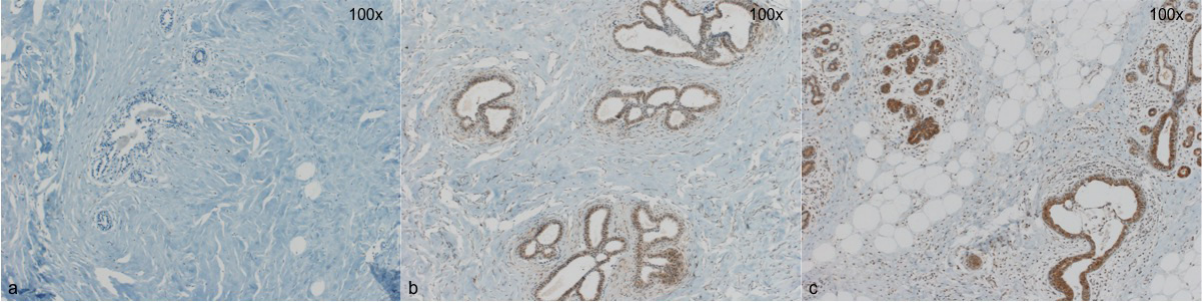
Three representative cases for Versican staining **a**: No detectible Versican expression in breast epithelial cells (0) **b**: Faint, predominantly nuclear Versican expression (1+) **c**: strong, cytoplasmic Versican expression (3+). Magnification 100x.

**Figure 2:**
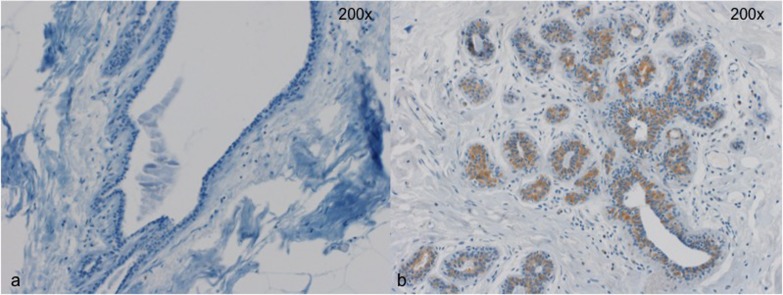
Two representative cases for Wnt4 staining **a**. No detectible Wnt4 expression in breast epithelial cells **b**. Wnt4 expression in a subset of epithelial cells. Magnification 200x.

**Figure 3:**
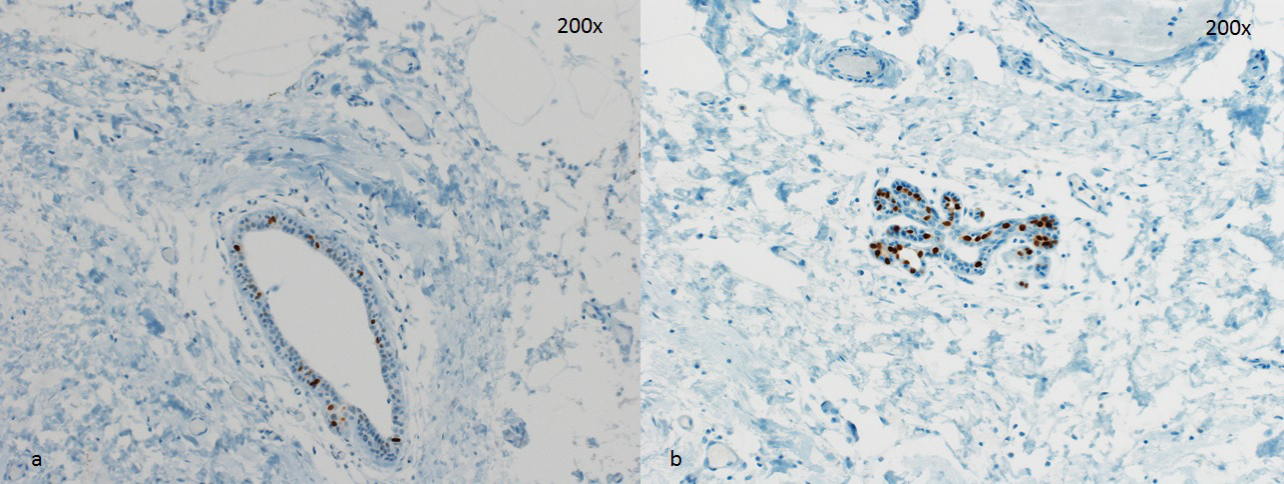
Two representative cases for PR staining **a**. few PR-positive cells in a lactiferous duct. **b**. lobule with almost all epithelial cells with PR expression. Magnification 200x.

**Figure 4:**
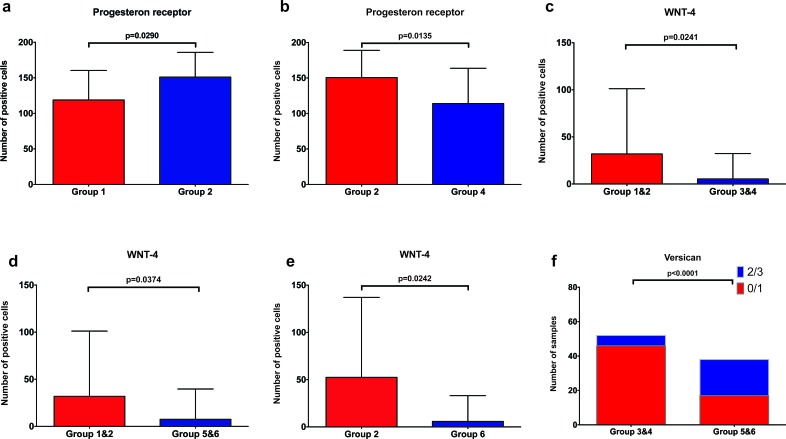
Graphic depiction of PR, Wnt4 and Versican expression between different subgroups Group 1&2: nulliparous women, Group 3&4: women with a full term pregnancy before the age of 30 years (early pregnancy), Group 5&6: women with a full term pregnancy after the age of 30 years (late pregnancy), Group 2: nulliparous women with subsequent breast cancer, Group 4: women with early pregnancy and no subsequent breast cancer. **a**. significantly lower numbers of PR+ epithelial cells in nulliparous women without subsequent breast cancer (Group 1) compared to nulliparous women with subsequent breast cancer (Group 2) **b**. significantly lower numbers of PR+ epithelial cells in women with early pregnancy and no subsequent breast cancer (Group 4) compared to nulliparous women with subsequent breast cancer (Group 2) **c**. significantly lower numbers of Wnt4+ epithelial cells in women with early pregnancy compared to nulliparous women. **d**. significantly lower numbers of Wnt4+ epithelial cells in women with late pregnancy compared to nulliparous women **e**. significantly higher number of Wnt4+ cells in nulliparous women with subsequent cancer (Group 2) when compared to the late pregnancy group without subsequent breast cancer (Group 6) **f**. significantly lower Versican expression in epithelial cells in women with early pregnancy (Group 1&2) compared to women with late pregnancy (Group 5&6).

To determine if the pattern of expression of PR, Versican, Wnt4, CK15 and β-Catenin in benign breast tissues differed between the groups, we performed an immunohistochemical analysis for these markers. Representative staining for Versican, Wnt4 and PR are shown in Figures 1 to 3. For nulliparous women, the number of PR positive epithelial cells was significantly higher in the subgroup with subsequent breast cancer compared to those without subsequent cancer (*p* = 0.029, Mann-Whitney U test) (Figure 4a). Additionally, we observed that the number of PR positive epithelial cells was significantly higher in the group of nulliparous women with subsequent invasive breast cancer (Group 2) compared to the group of women with early pregnancy and no subsequent breast cancer (Group 4, *p* = 0.0135, Mann-Whitney U test) (Figure 4b). There was also a trend toward higher numbers of PR positive cells in the nulliparous women with subsequent cancer (Group 2) when compared to the women with late pregnancy and no subsequent cancer, but it did not reach statistical significance (Group 6, *p* = 0.0705, Mann-Whitney U test, data not shown).

Within the group of women without subsequent breast cancer, we detected a significantly higher number of Wnt4-positive cells in the group of nulliparous women (Group 1) compared to the group of women with early pregnancy (Group 4, *p* = 0.0449) (Supplementary Figure 1). Interestingly, there was also a significantly higher number of Wnt4-positive cells in the nulliparous group with subsequent breast cancer (Group 2) when compared to the early pregnancy group without breast cancer (Group 4, *p* = 0.0229, Mann-Whitney U test, Supplementary Figure 1). Overall, independent of subsequent breast cancer, the number of Wnt4-positive cells was significantly higher in nulliparous women (Groups 1 and 2) compared to women with early pregnancy (Groups 3 and 4, *p* = 0.0241, Mann-Whitney U test, Figure 4c), and compared to women with late pregnancy (Groups 5 and 6, *p* = 0.0374, Mann-Whitney U test, Figure 4d). Additionally, a significantly higher number of Wnt4-positive cells in nulliparous with subsequent cancer group (Group 2) when compared to the late pregnancy group without subsequent breast cancer was found (Group 6, *p* = 0.0368, Mann-Whitney U test) (Figure 4e).

For the Wnt target Versican, there was a significantly lower expression in the group of women with early pregnancy (Groups 3 and 4) compared to the women with late pregnancy (Groups 5 and 6, *p* < 0.0001, Fisher's exact test) (Figure 4f). Within the subgroups of women without subsequent breast cancer, Versican expression was also significantly lower in women with early pregnancy (Group 4) compared to women with late pregnancy (Group 6, *p* < 0.0001, Fisher's exact test) and compared to nulliparous women (Group 1, *p* = 0.0028, Fisher's exact test) (Supplementary Figure 1).

No association between PR (on a continuous scale) and Versican (on a continuous scale) in any of the individual groups could be found (*p* >0.05).

### For CK15 and β-Catenin, no significant difference in expression was detected between the different subgroups

We also analyzed 7 breast cancer cases (3 from Group 2/nulliparous women, 2 from Group 3/early pregnancy and 2 from Group 5/late pregnancy) with immunohistochemistry against PR, Versican, Wnt4, CD15 and β-Catenin. The number of cases was too small for a statistical analysis, but looking at the results there were no differences between the cancer cases. 3 breast cancers were completely negative for PR, and all but one were negative for Wnt-4 and Versican. It thus seems as if in these few available breast cancer cases, there was no particular activation of the Wnt-pathway.

## DISCUSSION

In the present study, we performed immunohistochemistry for PR, Wnt4, Versican, β-catenin and CK-15 on breast tissue from different subpopulations of women, stratified according to age at first pregnancy and occurrence of breast cancer. In accordance to correspondent mouse studies [17, 23], we found significantly more PR and Wnt4 positive epithelial cells in nulliparous women with subsequent invasive breast cancer compared to women with early pregnancy and no subsequent breast cancer. Furthermore, the number of Wnt4 positive cells in women with either early or late pregnancy was significantly lower when compared to that in nulliparous women, independent of subsequent breast cancer occurrence. By contrast, the expression of Versican in breast epithelial cells was significantly lower in women with an early pregnancy compared to nulliparous women and to women with a late pregnancy, also independent of subsequent breast cancer occurrence.

In accordance with the results of our study, PR positive cells are reduced not only in parous rodents, but also in parous humans [24, 26], when compared to nulliparous mice or humans. Despite the fact that in their study, Taylor et al. [24] analyzed breast tissue from women who had an induced abortion between the 5^th^ and 23^rd^ week of pregnancy, the reduction of PR positive epithelial cells was distinct when compared to nulliparous women. In their study, a possible association with subsequent breast cancer was not analyzed [24]. The results of our study indicate a significant impact of early pregnancy on the expression of PR in breast epithelial cells, supporting the results of the corresponding mouse study by Meier-Abt et al. [17, 23], who examined the cell-fate hypothesis on the level of gene expression in mice. Analogously, reduced Wnt signaling has also been found in parous women [25].

Meier-Abt et al. [17, 23] examined the cell-fate hypothesis on the level of gene expression in mice. They were able to identify a lifelong downregulation of the Wnt pathway after pregnancy, which may be caused via a downregulation of PR positive luminal cells in parous mice. Consequently, basal mammary stem/progenitor cells become less proliferative and epithelial cells show an increased differentiation phenotype [17], which may be the underlying mechanism for the protection of early pregnancy against breast cancer. Additionally, a decreased Wnt signaling has been found in parous women [25].

Our analysis of Wnt4 expression showed similarities to the results of PR. Additionally, expression of Wnt4 was significantly higher in nulliparous compared to parous women. However, since the number of cases evaluable for Wnt4 in our study was small (12 cases), further experiments to test this hypothesis are warranted.

By analyzing β-catenin, Versican and CK15, we examined important targets of the Wnt pathway. Although no significant difference was observed in the expression of β-catenin and CK15, a Wnt target and an epithelial stem cell marker, respectively, between the various groups, we found a clear impact of pregnancy and, interestingly, early pregnancy, on the expression of Versican, with significantly lower expression in women with early pregnancy compared to those with late pregnancy and to nulliparous women.

Our findings thus support the hypothesis that early pregnancy, possibly through a decrease of PR positive luminal cells, leads to a decreased activation of the Wnt- signaling pathway and subsequent lower expression of the Wnt target Versican, which, by inhibiting the proliferation of mammary stem/progenitor cells, may be the reason for the lower incidence of subsequent breast cancer in these women.

Nevertheless, the limiting factors of our study need to be addressed. First, the number of patients in each group is rather small and heterogeneous. Specifically, the number of cases that could be analyzed for Wnt4 was small. Despite the small sample size, we demonstrated a relationship between a higher expression of certain components of the Wnt pathway, such as PR, Wnt4 and Versican, and the occurrence of subsequent breast cancer.

## CONCLUSIONS

This is the first study examining the effect of early pregnancy on PR expression and the activation of the canonical Wnt/β-catenin pathway in human breast tissue. We demonstrated a lower expression of PR, Wnt4 and Versican in women with an early pregnancy compared to women with a late pregnancy and compared to nulliparous women. Our results suggest, in accordance with previous studies in mice, that the downregulation of epithelial Wnt signaling is associated with the protective effect of early pregnancy against breast cancer in humans. The reduction in Wnt signaling may occur through a decrease of PR-positive luminal cells, which results in a decreased expression of the PR target Wnt4 and the Wnt4 target Versican, and consequently in a decreased proliferation of stem/progenitor cells. The Wnt signaling cascade might thus represent a potential target for breast cancer prevention strategies and further studies to define the underlying molecular mechanisms are needed.

## MATERIALS AND METHODS

### Identification of patients

Based on the database of the Institute of Pathology at the University Hospital Basel and associated clinical information, a total of 125 women who underwent biopsy of benign breast tissue were identified. The cohort was divided into six groups based on age at first pregnancy and the occurrence of subsequent breast cancer (Table 1). Early pregnancy was defined as a full-term pregnancy before the age of 30, and late pregnancy as a full-term pregnancy after the age of 30. Additionally, 7 breast cancer cases from the subgroups with subsequent cancers were identified. The corresponding tissue blocks for each patient were collected from the archives, and new hematoxylin & eosin (HE) slides were prepared. All HE slides were reviewed by an experienced breast pathologist (S.M.), and one appropriate slide containing benign breast tissue was identified for each patient. The study was performed in accordance with the Swiss patient privacy laws and has been approved by the Ethikkommission Nordwest- und Zentralschweiz (EKNZ, proposal number 2014-397).

### Immunohistochemistry

From each appointed tissue block selected based on the HE slides, 4μm sections were cut. Immunohistochemical analyses were performed by using reagents specific for PR (Clone 790-2223, Ventana, prediluted), Versican (polyclonal, Booster, 1:50), Cytokeratin(CK)15 (Clone 28476, Abcam, 1:200), β-Catenin (Clone 760-4242, Ventana, prediluted) and Wnt4 (Clone 150596, Abcam, 1:50). For all immunohistochemical stainings, DAB was used as a chromogen. All antibodies were extensively tested and validated using normal breast tissue (progesterone), normal thyroid tissue (Wnt4), normal brain tissue (Versican), normal esophagus (CK15) and desmoid tumors (β-Catenin).

Immunohistochemical analyses were performed by R.M and S.M., blinded for the subgroups of the patients. For PR, Wnt4, CK15 and β-Catenin, 200 breast epithelial cells were counted and the number of positive cells for each marker was recorded. For Versican, the staining was scored as: 0 = absent staining, 1 = only nuclear staining, 2 = weak cytoplasmic staining, and 3 = strong cytoplasmic staining of epithelial cells. For statistical analyses, 0 andd were considered negative for Versican, and 2 and 3 (cytoplasmic staining) were considered positive. Examples of staining for Versican and Wnt4 are given in Figures 1 and 2.

### Statistical analysis

Statistical analyses for categorical and non-categorical variables were performed using Fisher's Exact and Mann-Whitney U tests. All tests were two-sided. *P*-values < 0.05 were considered statistically significant. All analyses were performed using Graphpad Prism 6.0 (Graphpad Software, Inc., La Jolla, CA) or SPSS v.20 (Endicott, New York, NY).

## SUPPLEMENTARY FIGURE



## References

[1] Torre LA, Bray F, Siegel RL, Ferlay J, Lortet-Tieulent J, Jemal A (2015). Global cancer statistics, 2012. CA Cancer J Clin.

[2] MacMahon B, Cole P, Lin TM, Lowe CR, Mirra AP, Ravnihar B, Salber EJ, Valaoras VG, Yuasa S (1970). Age at first birth and breast cancer risk. Bull World Health Organ.

[3] Albrektsen G, Heuch I, Hansen S, Kvale G (2005). Breast cancer risk by age at birth, time since birth and time intervals between births: exploring interaction effects. Br J Cancer.

[4] Institute NC (2003). Summary report: early reproductive events and breast cancer workshop.

[5] Trichopoulos D, Hsieh CC, Macmahon B, Lin TM, Lowe CR, Mirra AP, Ravnihar B, Salber EJ, Salber EJ, Valaoras VG, Yuasa S (1983). Age at Any Birth and Breast-Cancer Risk. International Journal of Cancer.

[6] Meier-Abt F, Bentires-Alj M (2014). How pregnancy at early age protects against breast cancer. Trends Mol Med.

[7] Ma H, Bernstein L, Pike MC, Ursin G (2006). Reproductive factors and breast cancer risk according to joint estrogen and progesterone receptor status: a meta-analysis of epidemiological studies. Breast Cancer Res.

[8] Britt K, Ashworth A, Smalley M (2007). Pregnancy and the risk of breast cancer. Endocr Relat Cancer.

[9] Rajkumar L, Kittrell FS, Guzman RC, Brown PH, Nandi S, Medina D (2007). Hormone-induced protection of mammary tumorigenesis in genetically engineered mouse models. Breast Cancer Res.

[10] Schedin P, Mitrenga T, McDaniel S, Kaeck M (2004). Mammary ECM composition and function are altered by reproductive state. Mol Carcinogen.

[11] Russo J, Balogh GA, Russo IH (2008). Full-term pregnancy induces a specific genomic signature in the human breast. Cancer Epidemiol Biomarkers Prev.

[12] Blakely CM, Stoddard AJ, Belka GK, Dugan KD, Notarfrancesco KL, Moody SE, D'Cruz CM, Chodosh LA (2006). Hormone-induced protection against mammary tumorigenesis is conserved in multiple rat strains and identifies a core gene expression signature induced by pregnancy. Cancer Res.

[13] Russo J, Tay LK, Russo IH (1982). Differentiation of the mammary gland and susceptibility to carcinogenesis. Breast Cancer Res Treat.

[14] Medina D (2005). Mammary developmental fate and breast cancer risk. Endocr Relat Cancer.

[15] Lindvall C, Bu W, Williams BO, Li Y (2007). Wnt signaling, stem cells, and the cellular origin of breast cancer. Stem Cell Rev.

[16] Wagner KU, Smith GH (2005). Pregnancy and stem cell behavior. J Mammary Gland Biol Neoplasia.

[17] Meier-Abt F, Milani E, Roloff T, Brinkhaus H, Duss S, Meyer DS, Klebba I, Balwierz PJ, van Nimwegen E, Bentires-Alj M (2013). Parity induces differentiation and reduces Wnt/Notch signaling ratio and proliferation potential of basal stem/progenitor cells isolated from mouse mammary epithelium. Breast Cancer Res.

[18] Angers S, Moon RT (2009). Proximal events in Wnt signal transduction. Nat Rev Mol Cell Biol.

[19] Roarty K, Rosen JM (2010). Wnt and mammary stem cells: hormones cannot fly wingless. Curr Opin Pharmacol.

[20] Willert K, Jones KA (2006). Wnt signaling: is the party in the nucleus?. Genes Dev.

[21] Li Y, Welm B, Podsypanina K, Huang S, Chamorro M, Zhang X, Rowlands T, Egeblad M, Cowin P, Werb Z, Tan LK, Rosen JM, Varmus HE (2003). Evidence that transgenes encoding components of the Wnt signaling pathway preferentially induce mammary cancers from progenitor cells. Proc Natl Acad Sci U S A.

[22] Lim SK, Lu SY, Kang SA, Tan HJ, Li Z, Adrian Wee ZN, Guan JS, Reddy Chichili VP, Sivaraman J, Putti T, Thike AA, Tan PH, Sudol M (2016). Wnt signaling promotes breast cancer by blocking ITCH-mediated degradation of the YAP/TAZ transcriptional coactivator WBP2. Cancer Res.

[23] Meier-Abt F, Brinkhaus H, Bentires-Alj M (2014). Early but not late pregnancy induces lifelong reductions in the proportion of mammary progesterone sensing cells and epithelial Wnt signaling. Breast Cancer Res.

[24] Taylor D, Pearce CL, Hovanessian-Larsen L, Downey S, Spicer DV, Bartow S, Pike MC, Wu AH, Hawes D (2009). Progesterone and estrogen receptors in pregnant and premenopausal non-pregnant normal human breast. Breast Cancer Res Treat.

[25] Choudhury S, Almendro V, Merino VF, Wu Z, Maruyama R, Su Y, Martins FC, Fackler MJ, Bessarabova M, Kowalczyk A, Conway T, Beresford-Smith B, Macintyre G (2013). Molecular profiling of human mammary gland links breast cancer risk to a p27(+) cell population with progenitor characteristics. Cell Stem Cell.

[26] Asztalos S, Gann PH, Hayes MK, Nonn L, Beam CA, Dai Y, Wiley EL, Tonetti DA (2010). Gene Expression Patterns in the Human Breast after Pregnancy. Cancer Prev Res.

