# Supplementary material for: Pregnancy at early age is associated with a reduction of progesterone-responsive cells and epithelial Wnt signaling in human breast tissue
Source: Oncotarget. 2017 Mar 8;8(14):22353–60. doi: 10.18632/oncotarget.16023 (PMC5410228; doi:10.18632/oncotarget.16023)
Supplement: Supplementary file 1 [file oncotarget-08-22353-s001.pdf]

Pregnancy at early age is associated with a reduction of progesterone-responsive cells and epithelial Wnt signaling in human breast tissue

Supplementary Material

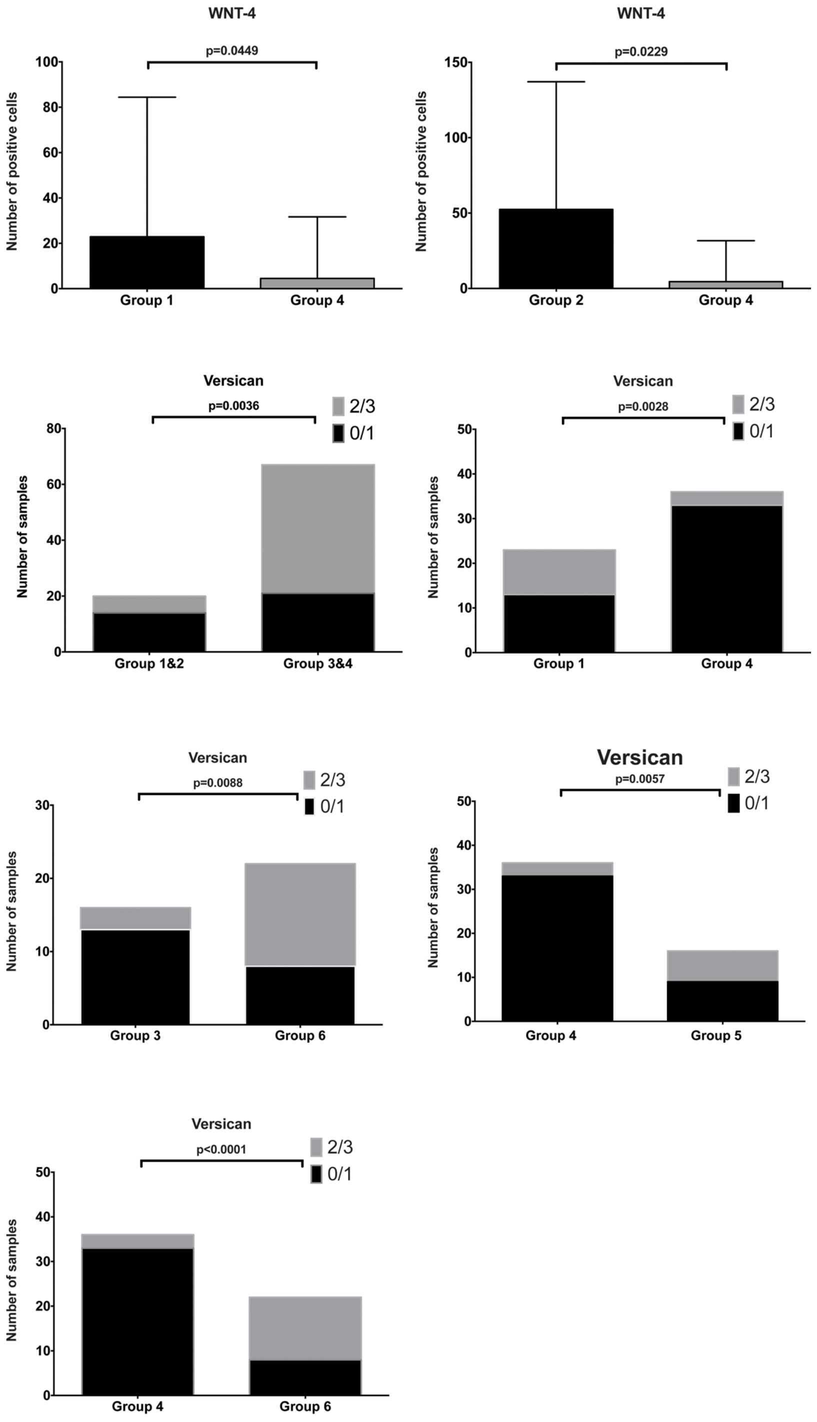

Supplementary Figure 1:
